# Impact of high-fat diet and exposure to constant light on reproductive competence of female ICR mice

**DOI:** 10.1242/bio.060088

**Published:** 2023-10-16

**Authors:** Kelsey Teeple, Prabha Rajput, Sara Scinto, Jenna Schoonmaker, Corrin Davis, Michayla Dinn, Mackenzie McIntosh, Sairam Krishnamurthy, Karen Plaut, Theresa Casey

**Affiliations:** ^1^ Department of Animal Science, Purdue University, West Lafayette, IN 47907, USA; ^2^ Neurotherapeutics Lab, Pharmaceutical Engineering and Technology, Indian Institute of Technology (Banaras Hindu University), Varanasi, UP 221005, India; ^3^ Histology Core, College of Veterinary Medicine, Purdue University West Lafayette, IN 47907, USA

**Keywords:** High-fat diet, Lactation, Light at night, Liver, Mammary, Maternal adaptions

## Abstract

Obesity and exposure to light at night are prevalent in modern society and associated with changes in physiology and behavior that can affect a female's ability to support offspring growth during pregnancy and lactation. A 2X3 factor study of ICR mice was conducted to determine the effect of diet [control (CON; 10% fat) or high fat (HF; 60% fat)] and exposure to regular 12 h light:dark cycles (LD) or continuous low (L5) or high (L100) lux of light on gestation length, birth litter size, milk composition and litter growth to lactation day 12. HF diet reduced birth litter size, but increased postnatal d 12 litter weight (*P*<0.05), whereas constant light tended to increase litter weight (*P*=0.07). Continuous light increased gestation length, altered dam feed intake, increased serum prolactin and increased final dam and mammary gland weight (*P*<0.05), while decreasing mammary ATP content and milk lactose (*P*<0.05). Correlation analysis indicated a positive relationship between final litter weight and mammary size, metabolic stores (e.g. maternal fat pad weight), kcal of feed intake, and gestation length (*P*<0.05). Although CON mice spent more time eating than HF dams, the calorically dense HF diet was related to greater rates of litter growth to peak lactation. Constant light circadian disrupting effects appear to be confounded by a potential long day photoperiod response exemplified by higher circulating levels of prolactin and increased body and mammary weight of females exposed to these conditions. Other model systems may be better to study the interacting effects of obesity and circadian disruption on reproductive competence.

## Teaser

**Summary:** High-fat diet and continuous light affected physiological and behavioral adaptions that occur in females to meet the metabolic and developmental needs of offspring during pregnancy and lactation.

## INTRODUCTION

In the modern era, working outside of the typical daylight hours has become common and results in exposure to continuous light (Lunde et al., 2020). Working overnight shifts and continuous exposure to light (light at night) disrupt circadian clocks that generate 24 h rhythms of physiology and behavior (Gatford et al., 2019). Circadian disruption has been linked to various ailments, including metabolic disruption and obesity, which is due to the inter-relationship between circadian clocks and metabolism (Bescos et al., 2018; Rumanova et al., 2020; Guan and Lazar, 2021).The modern age has also witnessed a drastic rise in obesity, especially in women of childbearing age (Hales et al., 2020; Driscoll and Gregory, 2020). The increased prevalence of obesity is likely the result of easy availability and palatability of energy-dense foods, and the rise in overnight work (Sarkar et al., 2019; Gatford et al., 2019). Obesity and circadian disruption in women have been linked to decreased fertility, poorer maternal health and lower lactation competence (Gatford et al., 2019; Casey et al., 2019, 2020).

To support fetal and neonate growth and development during gestation and lactation, females experience major alterations in metabolism and behavior that begin at the onset of pregnancy and continuously evolve to meet the changing needs of developing offspring (Speakman, 2008; Dai et al., 2011). Nutritional status and external environment impact hormonal milieu and metabolism, and thus impact the female's adaptive responses to the demands of pregnancy and lactation. Maternal metabolic adaptions create the nutritional environment of their offspring, which can program their long-term metabolic health (Augustine et al., 2008; Picó et al., 2021; Koletzko et al., 2019; Rodríguez-González et al., 2020). Maternal exposure to circadian disrupting environments during pregnancy and early lactation appears to negatively program offspring metabolism, as rat pups develop metabolic-like syndrome as adults (for example, Varcoe et al., 2011). Similarly, exposure to maternal obesity during gestation and lactation increases the risk of offspring developing metabolic syndrome as adults (for example, Picó et al., 2021).

The circadian system is highly integrated with both the reproductive and metabolic systems, with reciprocal regulation among them. Due to the close association with many other systems, such as the metabolic and endocrine systems, the disruption of circadian rhythms simultaneously disrupts these (Fishbein et al., 2021; Fatima and Rana, 2020; Potter et al., 2016). Circadian disruption not only alters hormonal and metabolic balance but also reproductive capacity of females (Kennaway et al., 2012; Potter et al., 2016; Fishbein et al., 2021). Exposing rat dams, ewes, and dairy cows to continuous changes in light:dark cycles to disrupt circadian clocks during gestation and lactation altered hormonal milieu and glucose homeostasis, and increased gestation length in cows and ewes (Varcoe et al., 2014; Varcoe et al., 2013; Suarez-Trujillo et al., 2022; McCabe et al., 2021).

**Fig. 1.**
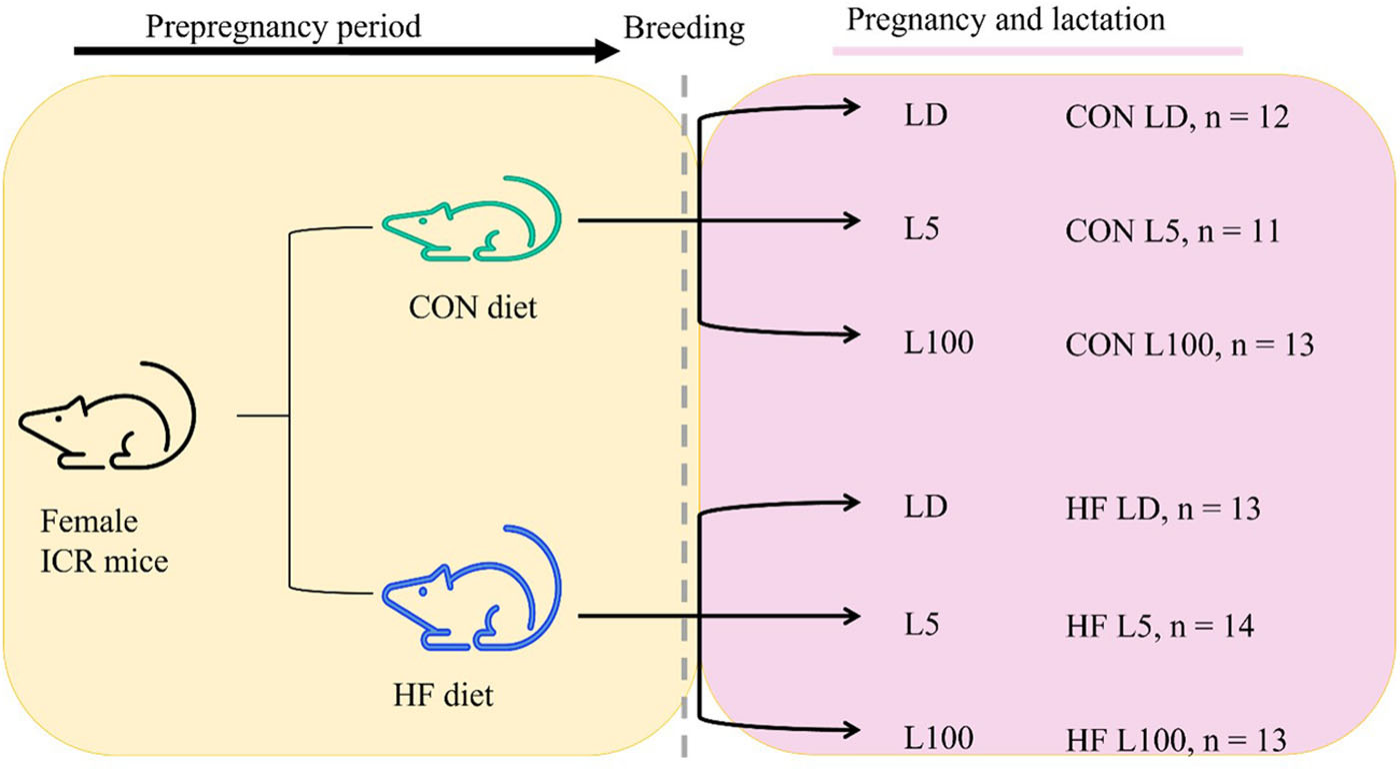
**Study design.** Female ICR mice were obtained from Enivgo (*n*=81) and assigned to control (CON; *n*=36) or high fat (HF; *n*=49) diet. After 4 weeks on diets, mice were bred. Upon observation of a vaginal plug or after 5 days, females were moved into one of three experimental light conditions: 12 h light:12 h dark (LD), continuous low lux light (L5), or continuous high lux light (L100). Mice remained on assigned diets and light treatments until euthanasia on day 12 of lactation.

Since a high-fat diet and exposure to light at night are increasingly common in modern society, our goal was to study how these factors alone or in combination affected female reproductive competence as defined by the ability to support fetal development during gestation and neonatal growth during lactation. Mouse models are commonly used to study biological phenomena that are relevant to human health, including feeding high-fat diets to induce obesity (Gao et al., 2015; Platt et al., 2014; Skaznik-Wikiel et al., 2016; West et al., 1992). We hypothesized that diet induced prepregnancy obesity and exposure to continuous light during pregnancy and lactation interact to affect maternal physiology and the dam's ability to support offspring growth during these reproductive stages. Our previous studies of the female ICR mice used in this study found altered eating behavior and attenuated fecal corticosterone circadian rhythms, that demonstrated high fat diet alone disrupted circadian rhythms in virgin female mice (Teeple et al., 2023). Here we describe findings of a two by three factor designed study (Fig. 1) that measured the impact of high-fat (HF) diet feeding prepregnancy and throughout pregnancy and lactation with and without exposure to a high lux (L100) or low lux (L5) of continuous light on gestation length, litter size at birth, milk composition, and litter growth to lactation day 12. The effect of treatment on maternal feed intake and weight change from mating to peak lactation, day 12, was also evaluated along with final weight of dam's liver, abdominal mammary glands, and urogenital fat pad, and measures of liver and mammary mitochondrial and oxidative phosphorylation taken to understand the impact of treatments on these maternal metabolic adaptions to lactation. We hypothesized that high fat diet and exposure to continuous light increased maternal stress, altered regulation of glucose homeostasis, and changed levels of photo responsive hormones. We, therefore, measured maternal hair corticosterone as a marker of physiological stress, glycated hemoglobin (HbA_1c_) levels to indicate maternal control of blood glucose, and serum prolactin as an indicator of response to abundance of light. Moreover, milk 8-hydroxy 2-deoxyguanosine was measured to assess oxidative damage to mammary mitochondrial DNA and malondialdehyde (MDA) to assess relative lipid peroxidation levels in the gland (Hadsell et al., 2011) to determine whether these were affected by diet or light exposures. We chose the outbred ICR strain of mice for the study due to their strong maternal characteristics, and based on the belief an outbred strain better encompasses the variability of the human population (Gao et al., 2015).

## RESULTS

### Effect of diet and light on feed intake during gestation, parturition, and lactation

At 5 weeks of age, nulliparous mice were assigned to a control (CON, 10% fat) or high-fat (HF, 60% fat) diet groups and allowed *ad libitum* intake for 4 weeks to achieve groups different in mass and body fat content prior to breeding (i.e. prepregnancy-diet induced obesity). Mice began the study at a similar weight (CON=21.7 g±0.52, HF=21.7 g±0.45), but at the completion of the 4-week premating period HF animals weighed more than CON animals (*P*<0.05). The mean weight of mice on HF diet was 26.60±0.39 g and those fed CON diet weighed 24.40±0.33 g diet. HF diet also significantly altered daily patterns of feed intake, fecal output and fecal corticosterone levels (see Teeple et al., 2023 for details).

**Fig. 2.**
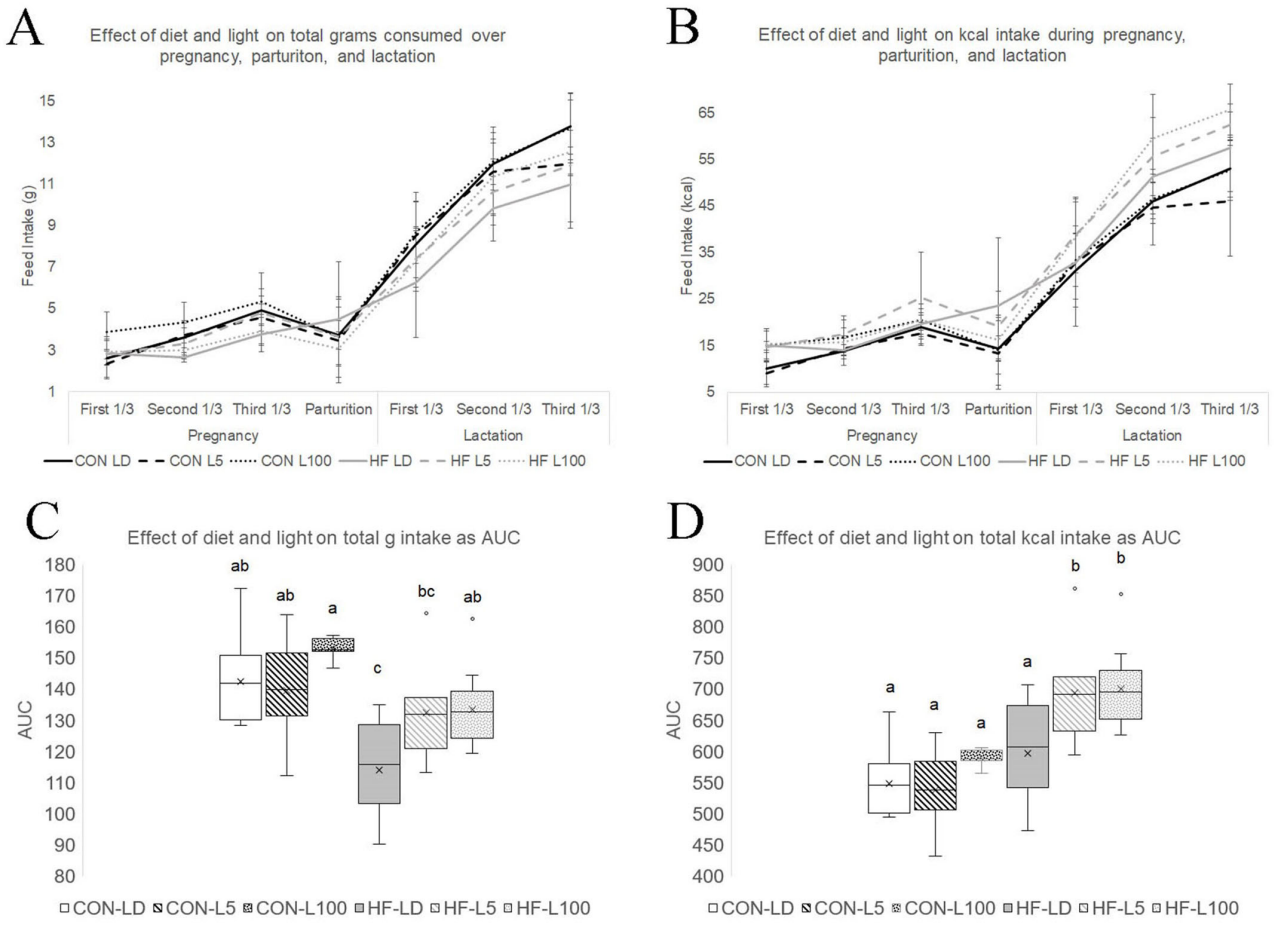
**Effect of diet and light on daily and total feed intake over pregnancy, parturition, and lactation.** Average daily feed intake was calculated over the experimental period as (A) mean grams (g)±SE and (B) mean kcal±s.e. taken in per day (d) for the first 1/3 of pregnancy (d1-d6), second 1/3 (d7-12), third 1/3 (d13- d prior to parturition), on day of parturition, and then d2-4, d5-8, and d9-11 of lactation. To determine total feed intake, the area under the curve (AUC) was calculated in (C) grams and (D) kcal. Differing letters indicant significant differences at *P*<0.05.

Females were mated with males at ∼9 weeks of age. After confirmation of breeding (observation of vaginal plug, pregnancy d1), animals remained on respective diets and were assigned to one of three light groups: LD, L5 or L100. LD animals remained on regular cycles of 12 h of light and 12 h of dark. Whereas L5 and L100 animals were exposed to continuous low lux (L5) or high lux (L100) of light. Dam feed intake was measured daily throughout the study, which ended on lactation d12. Diet (*P*<0.001) and stage of reproduction (i.e. pregnancy, parturition and lactation) significantly (*P*<0.001) influenced daily feed intake as measured in grams and kcal consumed per day (Fig. 2). Daily feed intake was the lowest at the beginning of pregnancy and increased until the end of lactation, with a dip at the time of parturition. CON mice consumed more grams (3.92±0.10 g) than HF (3.36±0.10 g), yet HF had higher average daily kcal intake (17.6±0.47 kcal) than CON (15.1±0.47 kcal) during pregnancy (*P*<0.05). During lactation, CON mice continued to consume a greater number of grams (9.24±0.18 g) each day compared to those on HF diet (8.27±0.17 g) and HF consumed more kcal (43.4±0.76 kcal) than CON (35.6±0.82 kcal).

There was a significant interaction between diet and light during pregnancy for grams of diet consumed (*P*=0.001) and kcal consumed (*P*=0.002) per day. Tukey post-hoc analysis demonstrated that CON-L100 mice consumed more grams (4.48 g±0.17) than CON-LD (3.73±0.18 g), CON-L5 (3.54±0.17 g), HF-LD (3.10±0.20 g), HF-L5 (3.64±0.16 g), and HF-L100 (3.32±0.17 g). CON-L100 consumed more kcal (17.2±0.81 kcal) than CON-L5 (13.6±0.79 kcal). CON-L5 mice also consumed less kcal than HF-L5 (19.1±0.73 kcal) and HF-L100 (17.3±0.78 kcal). Lastly, CON-LD mice consumed less (14.4±0.84 kcal) than HF-L5 mice (19.1±0.73 kcal). The significant diet and light interaction did not continue into lactation.

The area under the curve (AUC) for grams and kcal intake was calculated to estimate total amount consumed per mouse during pregnancy and from parturition to lactation day 12, and the sum of pregnancy and lactation (Fig. 2C,D). There was an effect of diet (*P*<0.001), a trend for light (*P*=0.08), and a significant interaction between diet and light (*P*=0.012) found for the AUC of grams of feed intake during pregnancy. AUC of kcal intake during pregnancy was significantly affected by diet (*P*=0.003) and diet and light showed a significant interaction (*P*=0.021). Diet and light had significant effects (*P*<0.05) on both AUC of total kcal and grams of intake across both pregnancy and lactation.

### Effect of diet and light on body and tissue weights

By lactation day 12, the difference in weight between CON and HF fed mice was lost, and there was no effect of diet on final weight of dams (Table 1). However, diet significantly influenced dam BMI (*P*<0.05), with HF mice having a higher BMI (4.77±0.07) compared to CON mice (4.54±0.07). At the time of assignment to light exposure for the study, there was no difference in weight of mice between LD, L5 and L100 groups. However, light had an overall effect on final dam weight, with animals exposed to continuous light weighing more than those exposed to LD. Light also affected (*P*<0.05) final mammary weight, with L100 mice having heavier glands.

**Table 1.**
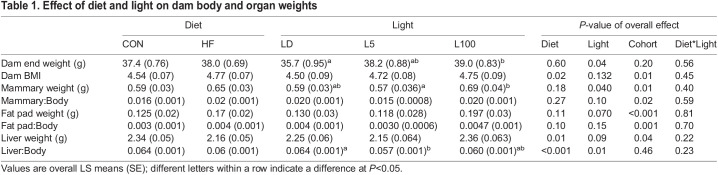
Effect of diet and light on dam body and organ weights

There was no effect of diet on fat pad weight, although the effect of light did approach significance at *P*=0.065, with weight of fat pad being greatest in L100 group. Neither diet (*P*=0.095) nor light (*P*=0.152) had an effect on the ratio of fat pad to body weight (Table 1). Diet impacted liver weight, with CON mice having larger livers (2.34±0.052 g) than HF mice (2.16±0.05 g). The ratio of dam liver to body weight was influenced by diet (*P*<0.001) and light (*P*=0.005). CON LD had the largest liver:body (0.069±0.002), whereas HF L5 (0.055±0.002) and HF L100 (0.055±0.002) mice had the smallest (Table 1). A significant cohort effect on final organ weights was also found, with animals in cohort 1 having heavier wet weights of mammary, liver and fat pads. *F*-values and number of mice per treatment are available in Table S1.

### Effect of diet and light on maternal plasma triacylglycerol (TAG) and prolactin, whole blood HbA_1c_, hair corticosterone, and liver Oil Red-O staining

At the completion of the study on lactation day 12, there was no difference in dam plasma TAG levels among the treatments (Table 2). Diet and light also did not impact plasma levels of HbA1c, although there was a significant cohort effect (*P*<0.001). Correlation analysis indicated that plasma level of HbA1c was positively related to final dam BMI (*r*=0.32, *P*=0.04; *n*=43). Mice on HF diet had higher hair corticosterone (*P*<0.05) at euthanasia (11.0±0.56 ng/g of hair) than CON (9.01±0.59 ng/g of hair). However, there was no effect of light on hair corticosterone. Light had an overall effect on dam plasma prolactin levels (*P*=0.04), where L100 mice had higher prolactin (15,753±3880 pg/ml) than LD mice (2047±3880 pg/ml). Due to the variance in plasma prolactin level across the animals, log transformed data was also investigated. Log transforming the prolactin data strengthened the effect of light on plasma prolactin (*P*=0.017), and the tendency of an effect (*P*<0.1) was lost for diet and cohort on plasma prolactin. Oil Red-O staining was used to assess the relative accumulation of fat in liver tissue (Fig. S1). There was a tendency for diet (*P*=0.08) to affect staining level, with CON mice have numerically greater scores than HF. Light had no effect on Oil Red-O staining. *F*-values and number per treatment are available in Table S2.

**Table 2.**
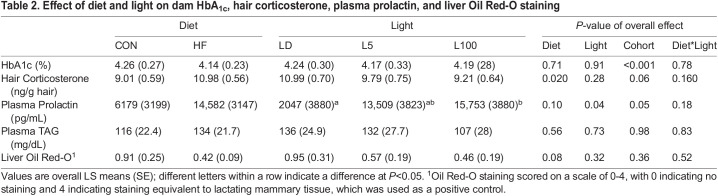
Effect of diet and light on dam HbA_1c_, hair corticosterone, plasma prolactin, and liver Oil Red-O staining

### Effect of diet and light on mitochondrial variables

Images captured of SDH staining activity demonstrate that in mammary tissue, levels were highest in the epithelium, which was selected to determine percent area of staining of this component of the gland (Fig. S2A). Whereas images of stained sections of the left lateral lobe of the liver demonstrate staining throughout tissue; areas lacking stain surround lobules and the central vein (Fig. S2B). The entire image captured was analysed to determine percent area of SDH staining in the liver tissue. In the mammary gland, percent area of epithelial SDH staining was not affected by diet nor light. In the liver, diet had no effect on SDH staining, but light had an overall effect (*P*=0.05; Table 3). *F*-values and number per treatment are available in Table S3.

**Table 3.**
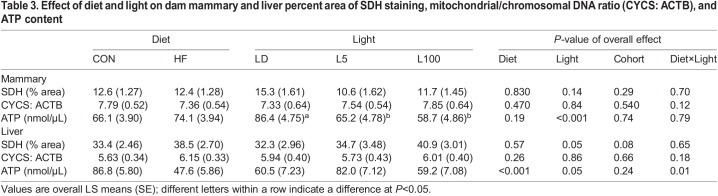
Effect of diet and light on dam mammary and liver percent area of SDH staining, mitochondrial/chromosomal DNA ratio (CYCS: ACTB), and ATP content

The ratio of the mitochondrial gene CYTB to the nuclear-chromosomal gene ACTB, was used as an indicator of relative number of mitochondria per cell. The ratio of CYTB:ACTB was not affected by diet nor light in either liver or mammary gland (Table 3). Although diet had no effect on mammary ATP content. Light exposure had an overall effect (*P*<0.001) on ATP content in the mammary gland (Table 3). Post-hoc analysis found that ATP content was lower in both L5 and L100 relative to LD treatment. Diet (*P*<0.001) and a trend for light (*P*=0.053) was found for the ATP content in the liver (Table 3). There was also a significant interaction of diet and light (*P*=0.005) in the liver. Post-hoc analysis indicated within CON fed mice, continuous light at L5 increased ATP content in liver. In the HF fed group, although not significant, there was numerically lower ATP content in liver of L100 versus LD. Pearson's correlation analysis found a strong positive relationship between SDH staining and ATP content in mammary (*R*^2^=0.92) and liver (*R*^2^=0.98).

### Effect of diet and light on milk composition

Neither diet nor light affected milk protein concentration on day 12 of lactation. Milk lactose was not affected by diet, but light had an overall effect on lactose content (*P*=0.003, Table 4). Mice exposed to continuous light had less lactose in milk than those exposed to LD. Levels of milk MDA were measured as an indicator of level of lipid peroxidation. MDA level was significantly greater in milk of HF versus CON mice. A significant interaction between diet and light was found for MDA levels, with HF diet and L100 increasing levels. Relative amount of DNA damage in mammary epithelial cells, as assessed by measuring 8-hydroxy 2′-deoxyguanosine (8-OHdG) in the milk (Hadsell et al., 2011), was not impacted by light, but was significantly affected by diet, with CON mice having elevated levels (1897±15.8 ng/mL) compared to HF mice (136±14.2 ng/mL). *F*-values and number per treatment are available in Table S4.

**Table 4.**
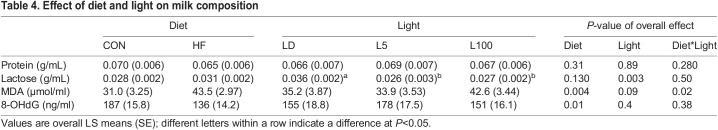
Effect of diet and light on milk composition

### Effect of diet and light on gestation length and number of pups born

**Fig. 3.**
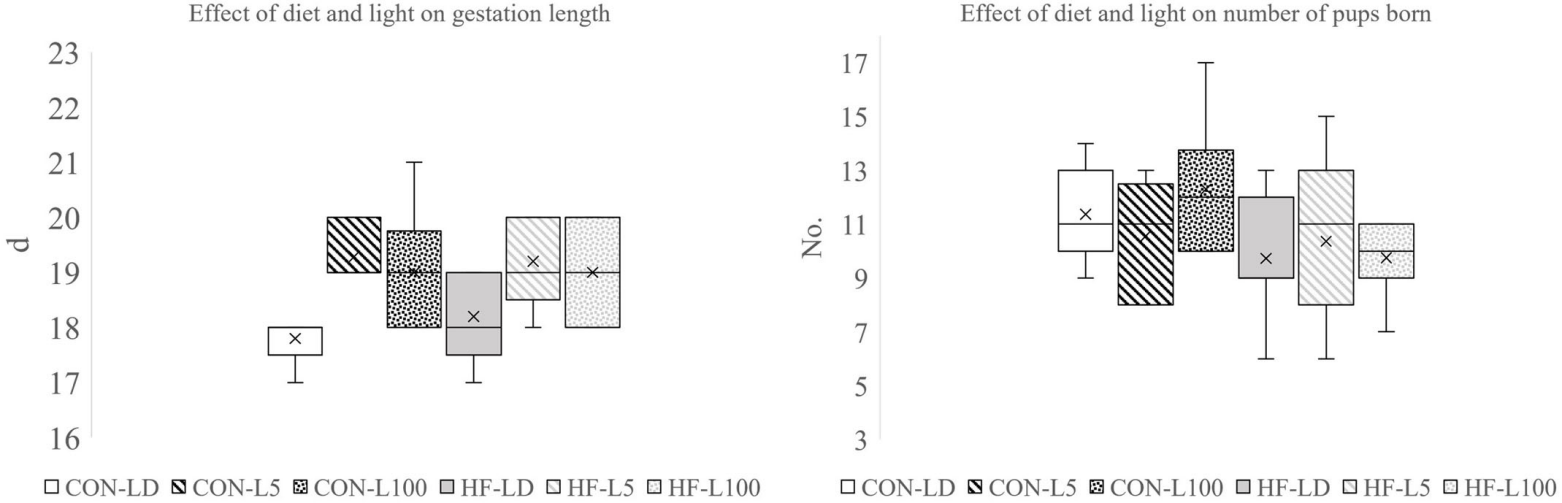
**Effect of diet and light on (A) gestation length and (B) birth litter size (number of pups).** Light had an overall effect on gestation length (*P*<0.05) with LD significantly lower than L5 and L100. Diet had an overall effect on birth litter size (*P*<0.05) with HF decreasing the number of pups born.

Although there was no effect of diet on gestation length, light had an overall effect (*P*=0.015; Fig. 3A). Post-hoc analysis demonstrated that L100 (18.9±0.21 d) and L5 (19.1±0.23 d) mice had longer gestations (*P*<0.05) compared to LD (18.1±0.25 d). There was no effect of light on number of pups born, but diet significantly impacted the number born (*P*=0.01), with CON mice having larger litters (11.4±0.4) than HF mice (9.99±0.4, Fig. 3B).

Pearson's correlation analysis of all maternal variables measured and number of pups born found maternal liver weight and ATP content positively related to birth litter size, whereas prepregnancy hair corticosterone levels and ratio of maternal fat pad weight to body weight were negatively correlated to number of pups born (Table 5). Within the HF group, a higher fat pad to body weight ratio was negatively related litter birth size. Similarly, within the LD cohort, milk MDA levels and fat pad weight were negatively related to birth litter size.

**Table 5.**
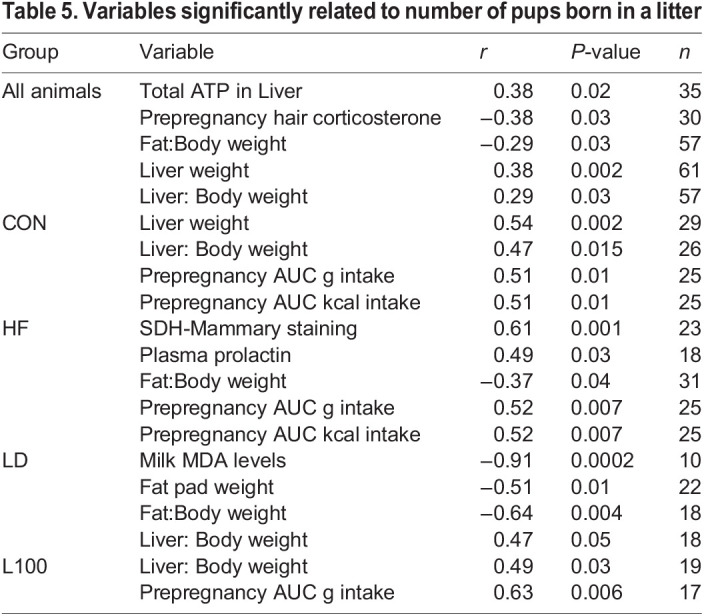
Variables significantly related to number of pups born in a litter

### Effect of diet and light on litter growth

**Fig. 4.**
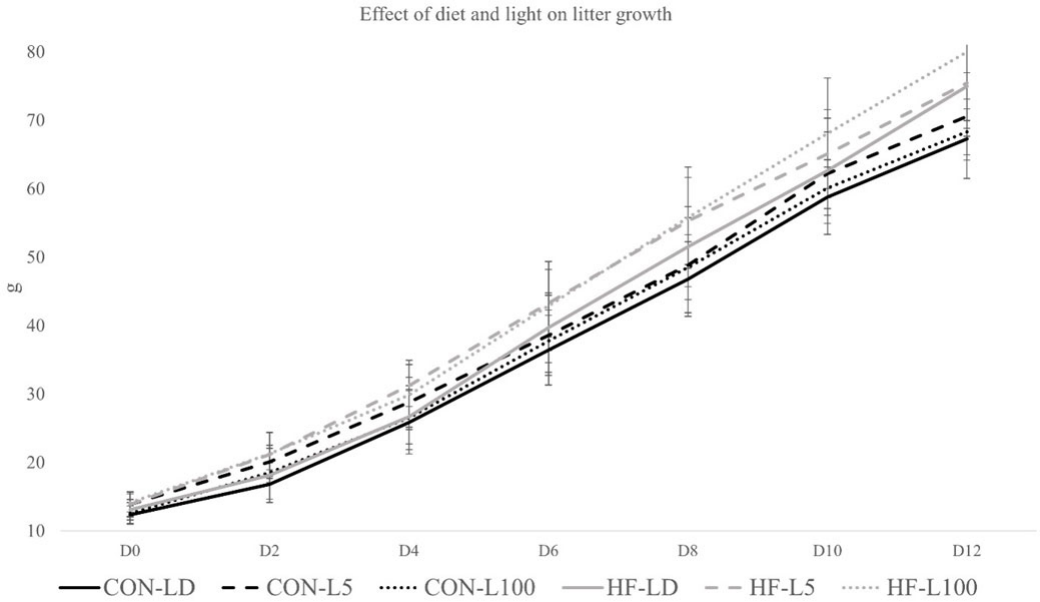
**Effect of diet and light on litter growth over 12 days of lactation.** Diet had an overall effect (*P*<0.05) on litter weight beginning on postnatal day 4 with HF litters weighing more than CON. Light had a tendency to have an effect (*P*=0.07) with continuous light increasing litter weight.

Litter growth was significantly influenced by diet, time, and cohort, and there was an interaction of diet and time (*P*<0.05; Fig. 4). Light did not significantly influence postnatal day 12 litter weight (*P*=0.07), but L5 and L100 treatments were numerically greater than LD within each diet group. Post-hoc analysis indicated no difference in standardized litter (*n*=8 pups) weights on day 0 or day 2, but HF litters were significantly heavier than CON beginning on day 4 (*P*=0.014) postnatal (Fig. 4).

Pearson's correlation analysis of all maternal variables measured and final litter weight found it was primarily related to variables that reflected dam and mammary size, metabolic stores (fat pad and BMI) and kcal of feed intake (Table 6). Gestation length was also positively correlated with litter weight. Across all dams, liver ATP content and AUC of g of feed intake during pregnancy were negatively correlated with litter weight on lactation day 12.

**Table 6.**
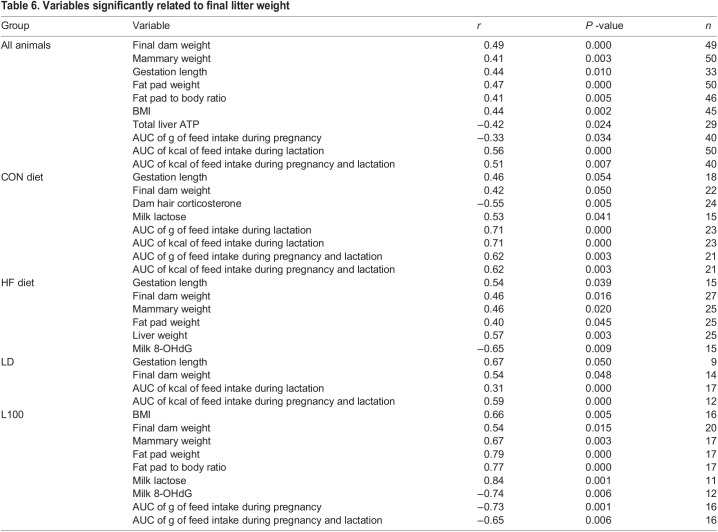
Variables significantly related to final litter weight

Analysis of relationships between dam variables and final litter weight within groups found that litter weight was positively associated with dam and mammary weight, feed intake in grams and kcal, gestation length, and milk lactose content within animals on CON diet. Hair corticosterone level was negatively related to final litter weight in CON diet cohort. Within the HF cohort gestation length, dam weight and mammary and fat pad weights as well as liver weight were positively related to final litter weight, but there was no relationship to feed intake. The level of 8-OHdG in milk, a marker of mammary DNA damage, was negatively related to litter weight on day 12 in HF dams. Within the LD group, final dam weight and feed intake were positively related to litter weight. Whereas in the L100 group dam, mammary and fat pad weight as well as BMI and lactose were positively related to final litter weight. Litter weight within the L100 cohort was negatively related to AUC of g of intake.

## DISCUSSION

Coordinated changes in metabolism and behavior occur to support the nutrient and energetic demands of the growing fetuses during pregnancy and milk production during lactation. Changes include increased feed intake, increased storage and then mobilization of fat and protein, and increased organ size, such as the liver and pancreas (Speakman, 2008; Johnson et al., 2001). Maternal adaptations to pregnancy and lactation can be modified by the female's environment and nutrition. The goal of this study was to investigate the effect of diet induced obesity and constant light on some of these metabolic adaptations in relation to litter size at birth and growth during lactation. We found profound effects of diet and light on maternal variables that reflected the manner females supported offspring growth and development during pregnancy and lactation. High fat diet consumption altered eating behavior prior to breeding, decreased number of pups born, limited maternal liver size, increased hair corticosterone levels, increased milk lipid peroxidation, and increased postnatal litter growth through peak lactation. Whereas continuous, bright light increased gestation length, dam and mammary weight, and plasma prolactin, but decreased mammary ATP content and milk lactose content.

Gestation length was increased in L5 and L100 groups relative to the LD treatment. Circadian clocks appear to play a central role in timing parturition. Studies of rodents found ablation of the maternal master clock resulted in altered parturition timing compared to rats with intact SCN (Reppert et al., 1987). When dairy cattle and sheep were exposed to circadian disrupting environments during pregnancy, gestation length was also increased (Gatford et al., 2019; Casey et al., 2022). Thus, we envision the increase in gestation length in groups exposed to continuous light was due to disruption to circadian rhythms that coordinate the timing of parturition.

Mice on HF diet had fewer pups born than dams on CON diet. Across all animals in this study, birth litter size was negatively related with prepregnancy hair corticosterone levels and the ratio of fat pad weight to body weight, an indicator of overall fattiness and comparable to BMI in humans. Correlation analysis of factors within the HF treatment similarly indicated a negative relationship between ratio of fat pad weight to body weight, BMI and litter size. Whereas in the LD group, milk MDA level was negatively related to birth litter size. The negative relationship between fat pad weight to body weight ratio, BMI with birth litter size reflects the association between obesity and infertility. Mice fed HF diets to induce obesity have decreased fertility related to a decreased number of primordial follicles produced in the ovary and altered estrous cycles (Wu et al., 2014; Brothers et al., 2010; Shaw et al., 1997). In humans and animal models, obese individuals often develop chronic inflammation and associated oxidative stress, and these states are believed to underly reduced fertility as oxidative stress damages oocytes and affects mitochondrial function and uterine environment (Snider and Wood, 2019). Animals on the HF diet had elevated milk MDA levels, a marker of lipid peroxidation, which reflects oxidative damage, and thus oxidative stress may underlie the smaller litter size of animals in this group. Additionally, there is potential that hypercortisolism caused embryonic loss in the HF group. Exogenous induction of hypercortisolism in female mice did not impact fertility if treatment ceased at the time of mating. However, when treatment continued into pregnancy, hypercortisolism caused a defective uterine environment, characterized by decreased implantation rates and fetal death (Li et al., 2018).

Human epidemiological findings and animal studies indicate that circadian disrupting environments cause irregular menstrual/estrous cycles, reduce ovulation rates and are related to embryonic and fetal loss (Hoshino et al., 2006; Casey et al., 2014; Boden et al., 2010; Bahougne et al., 2020; Gamble et al., 2013; Grajewski et al., 2015). Meta analysis of the impact of shift work on pregnancy in women found evidence for a slight increase in risk for preterm delivery, low birthweight, small-for-gestational-age infants and pre-eclampsia (Bonzini et al., 2011). We began continuous light exposure after mating, and thus lack of an effect on total number born indicates that the treatment did not cause early embryonic or fetal loss. Moreover, lack of a difference in standardized litter weight, suggest that continuous light did not affect *in utero* growth of pups.

While standardized litters (*n*=8 pups) did not differ in weight between diet or light treatments at birth, by day four postpartum, HF litters weighed significantly more than CON, which continued until day 12 of lactation. HF mice having heavier litters is consistent with our previous work (Chen et al., 2017). It is noteworthy to mention that although not significant, litter weight of groups exposed to continuous light showed a trend to be greater than the LD across most of lactation (*P*=0.07). We terminated the study on lactation day 12, because our interest was to gain an understanding of how maternal nutrition and light influenced litter growth. Around postnatal d10-12, rodent pups often begin to eat chow and open their eyes (Johnson et al., 2010), and thus growth begins to become independent of maternal influences. We envision, if the study could be continued beyond lactation day 12, litters of dams exposed to continuous light would be significantly larger than the LD group. Longitudinal studies of growth often show that trajectories initiated early in life continue into maturity (Rajia et al., 2010; Carmody et al., 2011; Devillers et al., 2011; Howie et al., 2009), and thus we believe that the effects of light are significant to the growth of litters.

Compared to non-pregnant states mice consume more feed during pregnancy and even more during lactation (Hammond and Diamond, 1992, 1994; Johnson et al., 2001). The increase in feed intake to support offspring growth is evident in current findings, with total feed intake increasing over the course of gestation and lactation, and a dip in feed intake at parturition. Diet significantly impacted both the number of grams and calories consumed each day. CON mice consumed a greater amount (g) of feed each day, whereas HF mice consumed a greater number of kcal. The need for CON animals to consume a greater number of grams reflects the need to meet the energy requirement necessary to support lactation versus the kcal rich HF diet. Additionally, at breeding, HF mice weighed more and had higher BMI and percent body fat than CON, reflecting a greater amount of fat stores (Teeple et al., 2023). The differences between HF and CON became diminished by the study's termination. These findings indicate HF dams mobilized their extra energy stores while CON mice had to obtain energy from increased food consumption to support litter growth (Augustine et al., 2008). Analysis of the relationship of feed intake to final litter weight indicated that across all mice the total number of kcal consumed during lactation and the entire study were positively related. There was also a positive relationship between dam weight and fat pad, indicating that maternal stores and feed intake are factors driving litter growth.

Calculations of area under the curve for total feed intake found that L100 mice on CON diet and L5 and L100 on HF diet consumed a greater number of grams of feed over the entire study period. In a companion study, we found that the greater number of grams were consumed regardless of time of day (no difference between 0600–1800 day or 1800−0600 night) in the continuous light groups, indicating disrupted behavioral patterns of eating. Within the HF group, the greater amount of eating of the animals exposed to continuous light resulted in consumption of a greater number of kcal in the L5 and L100 groups versus LD. The greater kcal intake resulted in L100 mice weighing more than LD at the time of euthanasia. A study that examined the effect of HF diet and constant light on estrous cycles of female Balb/C mice found that constant light and high fat diet caused greater weight gain over the 9 week study, with constant light having a greater effect than high fat diet (Zhang et al., 2021).

Continuous light elevated basal plasma prolactin levels at peak lactation. Prolactin synthesis in lactotrophs is controlled by circadian clocks and thus exhibits a strong circadian rhythm (Freeman et al., 2000). In mammals, levels of prolactin production also exhibit a seasonal response based on photoperiod. During short day photoperiods, prolactin secretion is suppressed relative to long day photoperiods across multiple species to include dairy cattle (Auchtung et al., 2005), rodents and sheep (Stewart and Marshall, 2022; Dodge and Badura, 2001, 2002). We interpret the elevated prolactin levels in the continuous light treatments as a response to the overall amount of light, as opposed to circadian disrupting effects (Lincoln et al., 2003; Dardente, 2007). Melatonin was not measured in our study as a marker of photoperiod response, as ICR mice are homozygous for B6J *Hiomt* mutant alleles, rendering them unable to produce melatonin and thus melatonin deficient (Kasahara et al., 2010). The elevation of circulating prolactin levels in animals exposed to continuous light is assumed to be accompanied by other changes in physiology related to long-day photoperiod which include alterations in metabolism and growth. Photoperiod studies of rodents have demonstrated that growth is elevated when animals are exposed to long day photoperiod conditions and growth is suppress under short day photoperiods (Tavolaro et al., 2015; Adam et al., 2000; Francisco et al., 2004). Thus, the larger size of the L5 and L100 animals may potentially be due to a growth response to total amount of light. Raising female laboratory rats in constant light increased circulating prolactin and estrogen levels relative to those exposed to regular LD cycles. Pubertal mammary development appeared precocious in animals exposed to constant light, with stage specific increased numbers of terminal end buds and alveolar buds and a higher rate of DNA synthesis (Mhatre et al., 1984). Melatonin treatment blocked these effects on mammary development, supporting a photoperiod effect. Similar to response of rats to continuous light, we found the wet weight of abdominal mammary glands of L100 animals was greater than LD mice. However, there was no difference in mammary gland:body ratio, suggesting that mammary gland growth was proportional to the change in body size.

Although mitochondrial number was not affected by light or diet, continuous light exposure, specifically L100 postpartum, decreased ATP content in the mammary gland relative to LD mice. Final dam and mammary gland weight were greater in mice exposed to continuous light than LD mice. The negative relationship between mammary size and ATP concentration illustrates how the mammary gland supports lactation. Continuous light increased body and mammary size. Mice exposed to LD had smaller body size and mammary glands and compensated for the likely fewer cells in the gland, partly, by producing more ATP. Moreover, the ATP content in liver of animals fed a HF diet was lower than those on CON diet. Litter weight on day 12 was negatively related to liver ATP content. The findings support that similar to mammary glands, there is an advantage of not depending on ATP production by the organ to support the demands of lactation.

The liver of HF dams weighed significantly less than those on CON diets. The liver nearly doubles in size from a nonpregnant state to day 18 of lactation in ICR mice (Bartlett et al., 2021; Dai et al., 2011). Hepatic growth during gestation resembles that of hepatocytes during liver regeneration, which aims to maintain a constant liver to body size ratio (Michalopoulos, 2017). The smaller livers of HF mice, and lower liver weight to body weight ratio, demonstrates that HF diet impacted hepatic growth. Correlation of variables affecting litter growth rate within the HF cohort found that liver weight was positively related to litter weight on day 12. Studies of rodents found high fat diet decreased the ability of the liver to regenerate following hepatic resection (Inaba et al., 2015; Jiang et al., 2011; Zhao et al., 2019). Accumulation of fat, hepatic steatosis, impairs hepatic hyperplasia and liver function (Della Fazia and Servillo, 2018). Although rodents fed HF diets have been shown to accumulate fat (Qiu et al., 2022; Korach-André, 2020; Tsuru et al., 2020), the Oil Red-O staining of liver tissue indicated a trend for mice on CON diets to have higher fat content, which suggests that liver fat content was not underlying differences in organ size. HF dams also had elevated hair corticosterone, which may limit liver growth (Barbason et al., 1989; Satoh and Yamazaki, 1993). Within the CON diet a negative relationship was found between high hair corticosterone levels and litter weight on day 12, which in addition to its association with smaller birth litter size, suggests maternal physiological stress may underlie some of the reproductive outcomes observed.

Neither diet nor light affected milk protein concentration. Lactose was affected by light, with LD having more lactose than L5 and L100. Lactose synthesis displays daily rhythm, with lactose having elevated levels during the morning and is nearly 60% lower during the night (Plaut and Casey, 2012; Kuhn et al., 1980). In mice expression of *LALBA*, which encodes alpha-lactalbumin, a component of the enzyme lactose synthase, exhibits a 24-h rhythm (Casey et al., 2014). These findings suggest that that the effect of continuous light on circadian clocks decreased lactose synthesis. Within the L100 treatment and CON diet, milk lactose content was positively related to litter weight on postnatal d 12. The observation that within the L100 treatment total grams of feed intake during pregnancy negatively associated with litter weight, may reflect the greater circadian disruption due to more time eating during rest ours and in turn lower lactose output. More studies are needed to understand whether lactose is driving this association or if it is a marker or accompanied by other changes in milk composition or nutritional environment of the neonates that is affecting their growth.

Previous studies found that feeding a HF diet to ICR mice during gestation and lactation increased milk fat content as measured by creamatocrit (Lucas et al., 1978; Chen et al., 2017). We similarly attempted to measure milk fat content using creamatocrit as well as with a triacylglycerol (TAG) colorimetric assay. The creamatocrit assay found no effect of diet nor light on percent fat, and the TAG assay found levels significantly depressed in the HF group. Due to the limited amount of milk and variation, we were not able to validate results, and so we did not feel confident enough in the data to include them in the results section of the manuscript. In contrast to our previous findings, others reported high fat feeding during lactation depressed milk fat content and *de novo* synthesis of fatty acids (Wahlig et al., 2012). Fat is the most variable component of milk, and so varying results across labs, studies, and strain of mouse may not be surprising (Görs et al., 2009). Based on these findings and the correlation between dam body size, fat-pad size and mammary-gland weight on lactation day 12, we do not believe that the macronutrient content of the milk explains the variation in litter growth observed. We did not perform weigh-suckle-weigh in the current study, and so we could not assess whether treatments affected overall milk yield. In another study, we found that dams on HF diet yielded more milk per weigh-suckle-weigh bout. Milk yield is determined by the number of lactocytes and their metabolic activity (Casey et al., 2022; Capuco and Choudhary, 2020). The positive relationship between final mammary weight and litter size, supports the assumption that the larger the mammary gland, the more milk producing cells it contains, and a greater milk yield, and thus litter growth.

We also investigated the hypothesis that exposure to continuous light and high fat diet feeding would both increase oxidative stress and damage mitochondrial DNA and lipids in the mammary gland. To investigate this hypothesis, we assessed DNA damage by measuring content of 8-hydroxy 2-deoxyguanosine, and found levels were elevated in milk of CON versus HF mice. This finding suggest that mitochondria of CON animals are exposed to a greater level of oxidative stress, which is consistent with higher production of ATP in mammary tissue of CON dams. Lipid peroxidation was assessed by measuring MDA concentration, and we found that HF diet significantly increased levels and a significant diet by light interaction, in which CON-L5 mice had the lowest amounts of lipid peroxidation compared to all other treatments. Work in dairy cattle has demonstrated an association with MDA levels and milk yield in which cattle that produce more milk have elevated MDA concentration (Kapusta et al., 2018). Perhaps MDA concentration in our current study may be reflective of milk yield. CON-LD and CON-L5 mice had very similar litter weights on day 12 of lactation, while CON-L100 mice were numerically heavier. All HF litters were heavier than the CON counterparts. Therefore, the lower MDA concentrations and lipid peroxidation of CON-LD and CON-L5 mice may reflect lower milk production compared to CON-L100 and all HF groups. HF diets are also associated with peroxidation of lipids and associated pathologies (for example, non-alcoholic fatty liver disease and liver cirrhosis) (Martín-Fernández et al., 2022; Hayasaka et al., 2017; Jiang et al., 2022, 2011). Further studies are needed to understand the effect of maternal diet on lipid peroxidation and the potential consequence to offspring being exposed to elevated levels in milk.

There were several weaknesses in this study. Our overall goal was to investigate the effect of prepregnancy obesity induced by HF diet and continuous light on the reproductive competence of ICR mice to develop a model system to study these commonly occurring factors in modern human populations. We chose to use ICR mice to capture the variability that is commonly observed between humans. However, since we acquired mice over two cohorts and the backgrounds of ICR mice is variable, this potentially led to a large confounding cohort effect in the present study. Additionally, obesity is difficult to define in rodent models and increasing the length of time and the subsequent weight gain of the mice could result in severe consequences to fertility (Gao et al., 2015). Therefore, after 4 weeks on the HF diet, we decided to breed mice, even though obesity, as defined by 30% greater than median of CON, was not reached in majority of the mice. Moreover, the difference in weight that existed prior to mating between CON and HF was lost by lactation day 12. Women with prepregnancy obesity typically do not change BMI over the course of lactation and only lose minor amounts of weight (Marshall et al., 2022; Winkvist and Rasmussen, 1999), and thus the prepregnancy obesity aspect of the rodent model did not mirror typical outcomes in humans. The positive relationship found between HbA_1c_ and BMI measures of mice may indicate that states reflective of greater insulin resistance may be achieved in mouse models in reproducing states. Efforts toward more forward selection starting with larger cohorts of mice may help in the study of this phenomena. Lastly, it should be noted that CON diet was high in starch. Diets with high glucose content can lead to hyperglycemia, whereas low carbohydrate diets can alleviate the glycemic load, thus a lack of difference in HbA_1c_ between CON and HF may be due to differences in dietary content (Jung and Choi, 2017; Vlachos et al., 2020).

It is also important to highlight that the continuous light exposure increased the size and total mammary mass relative to the LD animals. Continuous light also increased circulating prolactin levels. We believe, because of the relative short generation time, that some of the responses to continuous light may be more reflective of responses to long day photoperiod responses rather than circadian disruption, and thus this model confounds interpretation of some of the data.

In conclusion, HF-diet feeding and constant light exposure of female ICR mice during gestation and lactation affected maternal adaptations to these reproductive states including alterations in feeding behavior. CON mice spent more time eating than HF to support the metabolic demand of pregnancy and lactation, and yet the calorically dense HF diet was related to greater rates of litter growth to peak lactation. Exposure to continuous light increased gestation length, yet HF animals had fewer pups at birth. There was a trend for litters of dams exposed to continuous light to be heavier on lactation day 12. Continuous light significantly increased dam weight, mammary weight, and total feed intake and these variables positively related to litter weight at peak lactation. Differences in milk composition by treatment, which was marked by lower lactose in continuous light treatments, could not explain treatment differences in litter weight. Thus, in ICR mice, a HF diet and increased dam and mammary size appear to the primary drivers of postnatal litter growth, which likely reflects a greater ability for milk production. Higher levels of ATP in mammary and liver tissue of dams were related to lower litter weights, indicating that oxidative phosphorylation in these organs cannot close gaps of energetic and substrate synthetic demands of lactation that likely greater mammary development allowed. Further studies are needed to understand the origin and consequence of higher levels of MDA in milk of HF fed mice. MDA is a marker of lipid peroxidation and thus likely reflective of oxidative stress and inflammation, and may be linked to smaller birth litter sizes and lower maternal liver weight in this group. Finally, study findings indicate that constant light may not be the best model system to study the impact of circadian disruption on gestation and lactation in mice. Constant light circadian disrupting effects appear to be confounded by a potential long day photoperiod response exemplified by higher circulating levels of prolactin and increased size of females exposed to these conditions. Other model systems like exposure to chronic light shifting may be better to study the interacting effects of obesity and circadian disruption on reproductive competence.

## MATERIALS AND METHODS

### Animals and treatments

Prior to the start of the study, animal use protocols were reviewed and approved by Purdue University's Institutional Animal Care and Use Committee (protocol number 2104002135). Three-week-old female ICR mice (*n*=87; CD1, Envigo, Indianapolis, IN, USA) were received, ear tagged, and permitted to acclimate for 2 weeks. Experiments occurred over two cohorts (cohort 1, *n*=42; cohort 2, *n*=43) of mice. Following the 2-week acclimation period, mice were randomly assigned to experimental diets: control (CON, *n*=36) or high fat (HF, *n*=49) (Fig. 1). More females were placed in HF group to account for expected decreased fertility (approximately 80% of CON females) (Tortoriello et al., 2004).

Mice in each dietary treatment started off at a similar weight (CON=21.7 g±0.52, HF=21.7 g±0.45) (see Teeple et al., 2023 for details). To achieve groups divergent in mass and fat content prior to mating, mice were housed with three to five animals per cage based on diets and fed for *ad libitum* intake for 4 weeks. The diets were matched for sucrose content (7% sucrose) but with 10% energy in fat, 20% energy from protein, and 70% energy from starch for CON (Research Diet #D12450J; 3.85 kcal/g) and 60% energy in fat, 20% for protein, and 20% for carbohydrate for HF (Research Diet #D12492; 5.24 kcal/g). CON diet fat composition consisted of 23.70 g of soybean oil per 1000 g of diet and 18.96 g of lard per 1000 g of diet, while HF diet had 32.31 g of soybean oil per 1000 g of diet and 316.60 g of lard per 1000 g of diet. The CON has 479.69 g of corn starch per 1000 g of food, whereas HF diet contained 0 g of corn starch. Full comprehensive components of experimental diets are available at Research Diets, Inc. as well as in our previous publication (Suarez-Trujillo et al., 2019).

After the 4 weeks on diets, hair was shaved to measure prepregnancy corticosterone accumulation (Teeple et al., 2023). Females were then placed with males in a two female to one male breeding scheme. Mice were checked for presence of vaginal plugs twice daily at 0600 and 1700. Once a plug was observed or after 5 days with the male, females were moved into one of three experimental light treatments: 12 h of bright light and 12 h of dark (LD), continuous dim light (L5), and continuous bright light (L100). The intensity of light was measured with a light meter (Thermo Fisher Scientific, MA, USA) inside a cage on the racks to determine the lux at the eye level of the mice. The average lux of light of the rooms was 114±13.78 lux in LD, 3±1.51 lux in L5, and 106.25±7.91 lux in L100.

Mice remained on experimental diets and in experimental light conditions until the end of study on day 12 of lactation. The study was designed to be terminated on day 12 of lactation, as this is when pups start to open their eyes and consume the dam's food. Studies of rodents indicate the master clock in the suprachiasmatic nuclei (SCN) of the hypothalamus are not fully developed until day 10 postpartum (PP10), and that rhythms of clock genes begin to show adult-like amplitudes only after PP10 (Sumová et al., 2006; Sumová et al., 2008; Cheng and Cheng, 2021). Prior to this age in rodents, perinatal circadian rhythms are entrained by maternal cues, and not photic cues.

Pregnancy day 1 was defined as the day vaginal plug was observed. Once females gave birth, the gestation length was determined by counting the number of days between the observation of a vaginal plug and the date of parturition. When pups were observed and the dam appeared to have finished giving birth, pups were counted to determine birth litter size. Within 24-48 h of birth, the total number of pups were counted and then litters were standardized to eight pups per dam and weighed. For litters under eight pups, neonates from a litter larger than eight were cross fostered onto the dam. For litters over eight neonates, pups were euthanized via decapitation. The entire litter was weighed every other day.

On day 12 of lactation, dams and pups were separated for at least 3 h prior to milking dam. Dam and pup separation started at 0600. Immediately after separation, the pup sex and individual weight were recorded. After the 3-h separation, dams were anesthetized using 3% isoflurane gas at a rate of 1.0 L/minute oxygen. Milk was collected from mice as described by DePeters and Hovey, with several modifications (Depeters and Hovey, 2009). Dams were injected IP with 0.2 ml of oxytocin (20 Units/ml, Vet One, Boise, ID, USA). The area surrounding the teat was dampened using 18-ohm water. A rubber stopper was placed in a 2 ml microcentrifuge tube with two 21 G X 1.5 in needles going through it. One needle hub is used to create suction on the teat to collect milk directly into the 2 ml tube, and the other needle hub was connected to tygon tubing connected to an electric pump (Swing Breast Pump, Medela, Baar, Switzerland). Following milk collection, dams were euthanized via slow fill CO_2_ inhalation, followed by cervical dislocation as a secondary method. The method of euthanasia is an American Veterinary Medical Association approved method (Association, 2020).

Immediately after euthanasia, blood was collected from the dam via cardiac punction and placed in an 7.5% EDTA K3 plasma separating tube (Covidien, Dublin, Ireland). Dams were then weighed, crown–rump length was measured to determine BMI. Hair was collected with Wahl Mini Pro corded trimmers (Wahl Clipper Corporation, Sterling, IL, USA) for analysis of corticosterone.

Following hair collection, mammary, liver, and adipose tissue were collected and weighed. Both mammary gland number 4 were collected, and wet weight was taken prior to snap freezing in liquid nitrogen. A section of mammary number 3 was placed in optimal cutting temperature (OCT) compound (Thermo Fisher Scientific Inc.) and cryopreserved using liquid nitrogen. Next, the urogenital fat pad was collected and weighed. Lastly, the entire liver was removed and weighed, then a portion of the left lateral lobe of the liver was cryopreserved in OCT solution and the remainder of the lobe was snap frozen.

### Feed intake

Feed intake was determined by measuring feed consumed during the day between 0600–1800, which was the light phase for the LD group, and during the night between 1800–0600, which corresponded to the dark phase of the LD group. Feed was weighed Monday–Friday starting at 0600 and 1745 on an individual mouse basis. The two timepoints were added together and reported as total feed intake. Pregnancy feed intake was expressed on a weekly basis, and lactation feed intake broken down from day 1–4, 5–8, and 9–12. Kcal intake was determined by multiplying g consumed by 3.85 kcal/g for CON, or 5.24 kcal/g for HF.

### Blood and plasma analyses

Glycated hemoglobin (HbA_1c_) has been validated as a reliable marker to measure glycemic control in mice (Feng et al., 2019; Han et al., 2008; Dan et al., 1997). Whole blood HbA_1c_ and plasma levels of TAG and prolactin were measured. HbA_1c_ was measured in whole undiluted blood collected in EDTA tubes using a commercial HbA_1c_ assay kit (Crystal Chem, Elk Grove Village, IL, USA). Plasma TAG was measured by preparing a 1:5 dilution using the Cayman Chemical kit. Plasma prolactin was measured in a 1:20 dilution of plasma using a commercially available ELISA kit (ab100736, Abcam, MA, USA). All assays were performed following the manufacturers’ instructions.

### Hair corticosterone extraction and analysis using liquid chromatography-tandem mass spectrometry (LC-MS/MS)

Corticosterone extraction from hair and measurement using liquid chromatography tandem mass spectrometry (LC-MS/MS) was done at Purdue University's Metabolite Profiling Facility in Bindley Life Sciences. Approximately 30 mg of hair was cleansed with 2 ml of isopropyl alcohol (IPA), then IPA was decanted. Hair was dried in a heated room at 32˚C before being added to a Precellys MK28 (Bertin Technologies, SAS, Montigny-le-Bretonneux, France) lysing tube and weighed. Samples were loaded into a Precellys homogenizing centrifuge and set to 6500 rpm, three cycles, with the length of cycle rotation set to 30 s, and the length of rest at 20 s. Following homogenization, 1 ml of methanol+ISTD (10 ng deuterated d8-corticosterone, Toronto Research Chemicals, Toronto, ON, Canada) was added to each sample and incubated overnight on a rocker at 4°C. The next day, samples were centrifuged, and the supernatant was collected. The supernatant was then dried using a Savant SPD 2010 speed-vac (Waltham, MA, USA). Once dry, the samples were resuspended using 50 µl of Amplifex Keto Reagent (AB Sciex, Framingham, MA, USA) and incubated at room temperature for 1 h. Following the incubation, 30 µl of ddH_2_O was added into each sample and centrifuged at 13,000 rpm for 5 m. The supernatant was transferred to an LC vial and analysed using an Agilent 6470 QQQ LC-MS/MS system (Agilent, Santa Clara, CA, USA) with a C18 column and water/ACNN+0.1% FA buffer.

### Frozen tissue staining for succinate dehydrogenase (SDH)

SDH staining was carried out as described by Hadsell et al. (2011). Frozen OCT blocks of liver and mammary tissue were cryosectioned between −15 and −20° C on the Leica CM1860 Cryostat (Leica Microsystems Inc., Buffalo Grove, IL, USA) at Purdue University Histology Research Laboratory, and 5-µm tissue slices were placed on glass slides (Hadsell et al., 2011). Frozen sections were incubated in a solution of 4 mg/ml nitro blue tetrazolium chloride, 0.2 M Tris, 0.05 M MgCl_2_, and 0.83 M sodium succinate (Sigma-Aldrich) for 1 h at 37°C. The sections were then transferred to 15% formol saline containing 0.9% w/v NaCl and 15% w/v paraformaldehyde and incubated for 15 min at room temperature. Sections were washed in distilled water for 3 min, dehydrated in 93%, 95%, and 100% alcohol for 3 min each. The sections were rinsed in xylene, mounted with two drops of DPX mounting medium (Sigma Aldrich), and cover slipped.

Four images of liver or mammary tissue sections per mouse were captured using Nikon Eclipse 50i microscope (Nikon Inc., New York, NY, USA; Evolution MP, Media Cybernetics Inc., Rockville, MD, USA) microscope using the 40X objective. Percent area of staining in each image was measured using Image J software. Prior to measuring area, images were converted to black (stained area) and white (absence of stain) using the eight-bit threshold tool.

### DNA Extraction and quantitative polymerase chain reaction to compare mitochondrial DNA to chromosomal DNA

To assess relative number of mitochondria per cell in mammary and liver tissue the ratio of the mitochondrial gene cytochrome B (CYTB) to chromosomal-nuclear DNA beta actin (ACTB) gene was measured (Hadsell et al., 2011). DNA was extracted from mammary and liver tissue using the gMAX Mini Genomic DNA kit (IBI Scientific, Dubuque, IA, USA) by following the manufacturer's instructions with some modification. Frozen samples were thawed on ice and approximately 10 mg of tissue was weighed and transferred to a 2 ml microcentrifuge tubes containing 200 μl GST buffer. Tissue was homogenized using the Fisherbrand 150 handheld homogenizer for 10–20 s. Between samples, the homogenizer was rinsed with ethanol followed by ddH2O. Homogenized samples were transferred to a new 1.5 ml microcentrifuge tube with 20 µl of proteinase K from the kit and incubated for 6 h in a 60°C water bath. Samples were vortex every hour during the 6 h incubation. The modification from the original protocol was, following incubation, samples were centrifuged for 2 min at 15,000×***g***, then supernatant was transferred to another 1.5 ml microcentrifuge tube and 200 µl of GSB buffer was added and vortex for 10 s. RNase A was added, samples were shaken vigorously, then incubated for 5 min at room temperature to ensure efficient RNA degradation. The remainder of the DNA extraction followed manufacturer's instructions.

DNA yield and purity was assessed via Nanodrop 2000 (Thermo Fisher Scientific). Acceptable OD260/OD280 were 1.9±0.03 and OD260/OD230 were 2.0±0.1. Real time quantitative PCR (RT-qPCR) was performed using 400 ng of isolated mammary DNA and 200 ng of liver DNA with the Bio-Rad CFX Connect Real-Time System (Bio-Rad, Hercules, CA, USA). TaqMan gene expression assays designed by Thermo Fisher to target the mitochondrial gene cytochrome B (CYTB; 4331182) and nuclear-chromosomal DNA beta actin (ACTB; 4352933E) were used for analysis.

A master mix containing 10 μl of Bullseye TaqProbe qPCR Master Mix (MidSci, St. Louis, MO, USA), 1 μl of the gene probe, and 4 μl of nuclease free water per sample was created for both genes. A final volume of 20 μl was loaded into the RT-qPCR machine, with 15 μl being the master mix for the genes and the remaining 5 μl being DNA. RT-qPCR initiated with denaturing at 95°C for 2 min, followed by 40 cycles of denaturing at 95°C for 15 s, and annealing at 60°C for 1 min. The ratio of mitochondrial DNA cycle threshold to genomic DNA was calculated and used as an indicator of relative number of mitochondria per cell (Hadsell et al., 2011).

### Analysis of total ATP content of liver and mammary tissue

Mammary and liver total ATP content was measured using Fluorometric ATP Assay Kit (ab83355, Abcam). Briefly, approximately 10 mg of tissue was washed in cold PBS, and then homogenized in 100 μl ice cold 2N perchloric acid (PCA) with a Dounce homogenizer (Thomas Scientific) using 10–15 passes. Samples were incubated on ice for 30–45 min, and then centrifuged at 13,000 ***g*** for 2 min at 4°C. Supernatant was transferred to a fresh tube, and volume was brought to 500 μl by adding ATP assay buffer.

PCA was precipitated by adding 100 µl of ice-cold 2 M KOH and sample was vortexed A 5 μl aliquot was used for testing using pH paper, and pH was adjusted to 6.5-8. pH by addition of 0.1 M KOH or PCA. Samples were centrifuge at 13,000 ***g*** for 15 min at 4°C and supernatant was collected and measured fluorometrically at Ex/Em=535/587 nm (Spark 10 M, and Plate: Thermo Fisher Scientific-Nunclon 96 Flat Black with transparent bottom, NUN96fb). The concentration was calculated as described in manufacturer's protocol and expressed as ng/µl.

### Oil Red-O staining and analysis of distribution in liver tissue

To evaluate the effect of diet and light treatment on fat content of liver, the fat-soluble stain Oil Red-O (Solvent Red 27, Sudan Red 5B) was used to stain neutral triacylglycerides and lipids in frozen sections. Frozen OCT liver blocks were cryosectioned between −15 to −20°C on the Leica CM1860 Cryostat (Leica Microsystems Inc.) at the Purdue University Histology Research Laboratory, and 5 µm tissue slices were placed on glass slides. Manufacturer's protocol for the Oil Red-O Stain Kit (StatLab, catalogue code KTOROPT, McKinney, TX, USA) were followed. Glass cover slips were applied with aqueous mounting media. Images from two areas of liver tissue were captured with the 20X objective using a Nikon Eclipse 50i microscope (Nikon Inc.). Oil Red-O staining was independently scored by two individuals trained by the same person. Staining was scored on a scale of 0–4, with 0 indicating no staining and 4 indicating staining equivalent to lactating mammary tissue, which was used as a positive control. Final scores were determined by averaging between the sections and across the reviewers.

### Milk composition analyses

Milk was thawed overnight at 4°C under constant rotation to homogenize. Milk protein was measured using a Bradford assay (Thermo Fisher Scientific) following diluting milk samples 1:100 in phosphate buffered saline (PBS). Lactose was measured using a lactose assay kit (MAK017, Sigma-Aldrich) and milk was prepared with a 1:500 dilution with 1X PBS. Other studies have demonstrated that measuring 8-hydroxy 2′-deoxyguanosine in milk reflects oxidative damage to DNA in the mammary gland (Hadsell et al., 2011). To measure milk 8-hydroxy 2′-deoxyguanosine (8-OHdG), an ELISA kit from Abcam (ab201734, Waltham, MA, USA) and milk was prepared as a 1:5 dilution. Milk malondialdehyde (MDA), which is a marker of lipid peroxidation, was prepared with a 1:5 dilution and measured using a colorimetric kit from Abcam (ab118970).

### Statistical analysis

The statistical analyses were conducted in R version 4.1.2 (Team, 2022). Significance was declared at *P*<0.05 and a tendency was 0.05≤*P*≤0.10. The afex package in R was used to analyse total feed intake in g and kcal, as well as litter growth over 12 days, as generalized linear mixed models (Singmann et al., 2022).

For total feed intake in g and kcal, there were five fixed factors: diet, light, term, the interaction between diet and light, and cohort. Term is the stage of reproduction (i.e. pregnancy, parturition and lactation) Mouse ID was the random variable in the feed intake models. For litter growth, the fixed effects were diet, light, day, and the interactions between diet and light, diet and day, light and day, and diet, light and day. In the litter growth model, the number of pups born was included as a potential confounding factor. The selection for our mixed models followed the processed outline by Singmann and Kellen, (2019). Based on the selection process, we selected the most complex model that would compute. The package emmeans was used for Tukey's post-hoc analysis, as well as to generate LS means (Lenth et al., 2022).

Dam end weight (g), dam BMI, mammary wet weight (g), the ratio of mammary gland to body weight, fat pad weight (g), the ratio of fat pat to body weight, liver weight (g), the ratio of liver weight to body weight, HbA_1c_, hair corticosterone (ng/g of hair), plasma prolactin (pg/ml), plasma TAG (mg/dl), gestation length (days), number of pups born, milk protein (g/ml), lactose (g/ml), milk MDA (µmole/ml), milk 8-hydroxy 2-deoxyguanosine (ng/ml), mammary and liver SDH staining (percent staining), ratio of mitochondrial and chromosomal DNA in mammary and liver, and ATP content of mammary and liver were all measured with general linear models to investigate the effects of diet, light, cohort, and the interaction of diet and light on the various measurements.

A correlation matrix was created using Pearson's correlation with the following variables: dam end weight, BMI, mammary weight, ratio of mammary to body weight, fat pad weight, ratio of fat pad to body weight, liver weight, ratio of liver to body weight, HbA_1c_, hair corticosterone, plasma prolactin, plasma TAG, gestation length, number of pups born, litter weight on day 12 of lactation, milk protein, milk MDA, milk 8-hydroxy 2-deoxyguanosine, mammary and liver SHD staining, ratio of mitochondrial to chromosomal DNA in mammary and liver, and ATP content of mammary and liver.

## Supplementary Material

10.1242/biolopen.060088_sup1 Supplementary information

Table S1. Number of observations (n), F-value (degrees of freedom, df), and P-value for the effect of diet and light on dam body and organ weights.

Table S2. Number of observations (n), F-value (degrees of freedom, df), and P-value for the effect of diet and light on dam HbA1c, hair corticosterone, and plasma prolactin

Table S3. Number of observations (n), F-value (degrees of freedom, df), and P-value for the effect of diet and light on mammary and liver SDH, mitochondrial/chromosomal DNA ratio, and ATP content

Table S4. Number of observations (n), F-value (degrees of freedom, df), and P-value for the effect of diet and light on milk composition
