# Supplementary material for: Impact of high-fat diet and exposure to constant light on reproductive competence of female ICR mice
Source: Biol Open. 2023 Oct 16;12(10):bio060088. doi: 10.1242/bio.060088 (PMC10602010; doi:10.1242/bio.060088)
Supplement: Supplementary information [file biolopen-12-060088-s1.pdf]

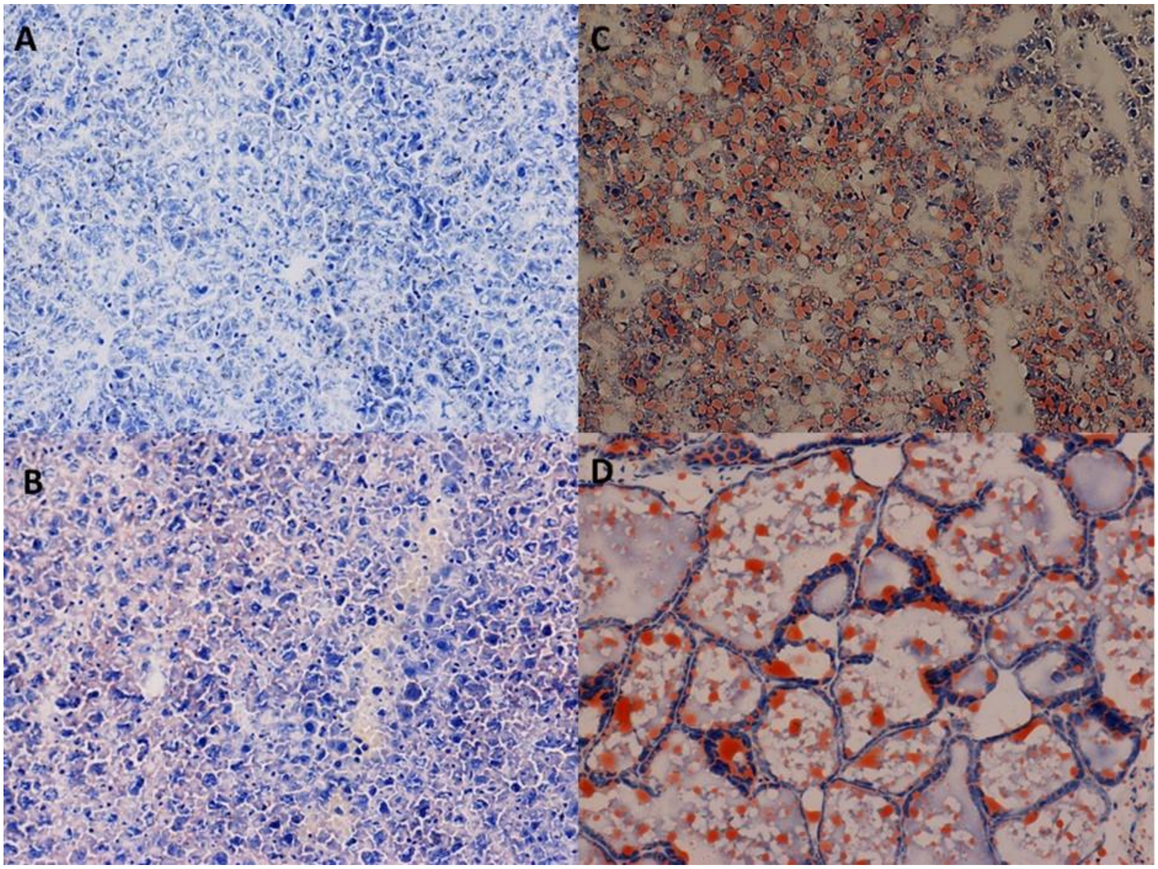

Fig. S1.

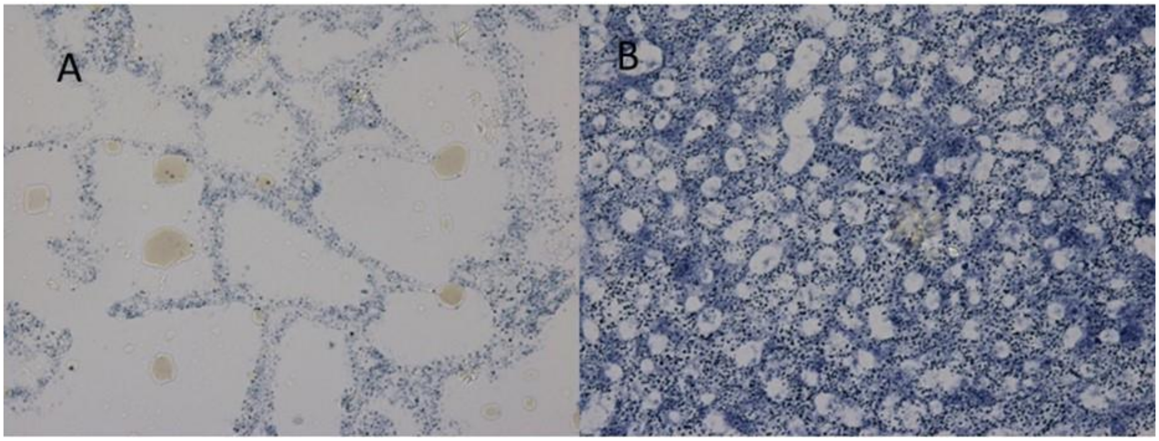

Fig. S2.

Table S1. Number of observations (n), F-value (degrees of freedom, df), and P-value for the effect of diet and light on dam body and organ weights.

Click here to download Table S1

Table S2. Number of observations (n), F-value (degrees of freedom, df), and P-value for the effect of diet and light on dam HbA1c, hair corticosterone, and plasma prolactin

Click here to download Table S2

Table S3. Number of observations (n), F-value (degrees of freedom, df), and P-value for the effect of diet and light on mammary and liver SDH, mitochondrial/chromosomal DNA ratio, and ATP content

Click here to download Table S3

Table S4. Number of observations (n), F-value (degrees of freedom, df), and P-value for the effect of diet and light on milk composition

Click here to download Table S4

Biology Open • Supplementary information
